# Recent Advances in Glioma Cancer Treatment: Conventional and Epigenetic Realms

**DOI:** 10.3390/vaccines10091448

**Published:** 2022-09-02

**Authors:** Mohsen Karami Fath, Kimiya Babakhaniyan, Mehran Anjomrooz, Mohammadrasoul Jalalifar, Seyed Danial Alizadeh, Zeinab Pourghasem, Parisa Abbasi Oshagh, Ali Azargoonjahromi, Faezeh Almasi, Hafza Zahira Manzoor, Bahman Khalesi, Navid Pourzardosht, Saeed Khalili, Zahra Payandeh

**Affiliations:** 1 Department of Cellular and Molecular Biology, Faculty of Biological Sciences, Kharazmi University, Tehran 1571914911, Iran; 2 Department of Medical Surgical Nursing, School of Nursing and Midwifery, Iran University of Medical Sciences, Tehran 1996713883, Iran; 3 Department of Radiology, Shariati Hospital, Tehran University of Medical Sciences, Tehran 1411713135, Iran; 4 Faculty of Medicine, Kerman University of Medical Sciences, Kerman 7616914115, Iran; 5 Department of Microbiology, Islamic Azad University of Lahijan, Gilan 4416939515, Iran; 6 Department of Biology, Faculty of Basic Sciences, Malayer University, Malayer 6571995863, Iran; 7 Department of Nursing, School of Nursing and Midwifery, Shiraz University of Medical Sciences, Shiraz 7417773539, Iran; 8 Pharmaceutical Biotechnology Lab, Department of Microbial Biotechnology, School of Biology and Center of Excellence in Phylogeny of Living Organisms, College of Science, University of Tehran, Tehran 1411734115, Iran; 9 Experimental and Translational Medicine, University of Insubria, Via jean Henry Dunant 3, 21100 Varese, Italy; 10 Department of Research and Production of Poultry Viral Vaccine, Razi Vaccine and Serum Research Institute, Agricultural Research, Education and Extension Organization, Karaj 3197619751, Iran; 11 Cellular and Molecular Research Center, Faculty of Medicine, Guilan University of Medical Sciences, Rasht 4193713111, Iran; 12 Department of Biology Sciences, Shahid Rajaee Teacher Training University, Tehran 1678815811, Iran; 13 Department of Medical Biochemistry and Biophysics, Division Medical Inflammation Research, Karolinska Institute, SE-17177 Stockholm, Sweden

**Keywords:** glioblastoma, cancer, brain tumor, treatment, epigenetic, peptide-based treatment, vaccine

## Abstract

Glioblastoma (GBM) is the most typical and aggressive form of primary brain tumor in adults, with a poor prognosis. Successful glioma treatment is hampered by ineffective medication distribution across the blood-brain barrier (BBB) and the emergence of drug resistance. Although a few FDA-approved multimodal treatments are available for glioblastoma, most patients still have poor prognoses. Targeting epigenetic variables, immunotherapy, gene therapy, and different vaccine- and peptide-based treatments are some innovative approaches to improve anti-glioma treatment efficacy. Following the identification of lymphatics in the central nervous system, immunotherapy offers a potential method with the potency to permeate the blood-brain barrier. This review will discuss the rationale, tactics, benefits, and drawbacks of current glioma therapy options in clinical and preclinical investigations.

## 1. Introduction

Gliomas are the brain’s most frequent and deadly tumors, accounting for roughly 30% of all brain malignancies [1,2]. Glioblastoma (GBM) is characterized by changes in cell metabolism, including an increased Warburg effect [3], dysfunctional oxidative phosphorylation (OXPHOS) [4], disrupted lipids metabolism [5], and other metabolic changes. The Warburg effect (also known as aerobic glycolysis) is one of the most important features of GBM. A group of glycolytic enzymes, including the HK1-3, PFK1, GAPDH, GLAM, ENO1, PKM2, and LDHA, regulates the Warburg effect [6,7]. Moreover, several novel signaling pathways and factors are also reported to be involved in the regulation of the Warburg effect, such as PI3K/Akt, LKB1/AMPK, HIF-1/2, p53, tyrosine kinases, and c-Myc [8,9].

Some glioma symptoms are mild and progressively worsen over time, while others are manifested as abrupt severe clinical symptoms [10,11]. The specific mechanism underlying glioma development is yet to be elucidated [12,13]. The World Health Organization (WHO) recently categorized central nervous system malignancies into four groups [14,15]. Gliomas are divided into four grades; the first two (grades I and II) are considered low-grade, and the last two (grades III and IV) are considered high-grade [16,17]. The degree of glioma malignancy grows with its grade [18]. Despite the fact that each grade has a precisely defined prognosis to guide clinical treatment, the majority of high-grade gliomas, such as grades III and IV anaplastic gliomas, lack any definitive treatment choices [19,20]. Surgery is the most common treatment for these high-grade tumors, followed by chemotherapy and radiation therapy [21]. Temozolomide (TMZ) and dozens of other treatments are currently used to treat these tumors [22]. TMZ is a chemotherapeutic medication capable of crossing the blood-brain barrier [23]. Aside from their numerous adverse effects and the poor prognosis, glioma resistance to radiation and chemotherapy is an additional concern [24,25].

One of the significant mechanisms of drug resistance is related to the activity of DNA-guanine methyl-O6 methyltransferase [26,27]. This enzyme can attach to an alkyl group in the O6 atom of guanine and decrease its availability [28]. Recent clinical studies have proposed targeted molecular therapy to overcome these restrictions [29,30].

In addition, genetic and epigenetic abnormalities are intimately correlated with the development and molecular pathology of malignant glioma [31]. These aberrations include abnormal methylation, microRNA changes, abnormal DNA, discoloration and structural changes, DNA methylation [32], and histone modifications (methylation, acetylation, phosphorylation, and other modifications) [33]. These changes play a crucial role in GBM development and pathology [34,35,36].

The various genes and enzymes of epigenetic regulations have now emerged as novel therapeutic targets for the treatment of gliomas and other malignancies [37]. Moreover, improving the immune responses to tumor antigens, rewiring the TME to prevent glioma-induced immunosuppression, and restoring normal angiogenesis could be achieved by gene therapy [38,39]. Gene therapy using nanoparticles is one of the prevailing strategies for glioma treatment [40].

This study summarizes the existing treatment options for glioma. Moreover, it elaborates the possible therapeutic approaches, based on immunotherapy, vaccination, epigenetics, viral vehicles, and peptide moieties. We explain how epigenetic processes control disease etiology in a dynamic manner. The clinical trials and their findings regarding epigenetic-based therapies for glioma are also described.

## 2. Epigenetics in the Pathogenesis of Glioma

### 2.1. DNA Methylation

DNA methylation patterns in glioma cells are reported to differ from normal cells [41,42]. Most significantly, tumor cells are characterized to have widespread hypomethylation and CpG island hypermethylation. Therefore, the methylation status of glioma-related genes could be a useful diagnostic factor. Given the differences in methylation patterns and the prognostic G-CIMP index of gliomas in adults and children, adult-specific indicators of glioma must be identified. Most CpG islands are hypomethylated under normal physiological conditions [43]. However, a tumor suppressor and DNA repair genes often cause hypermethylation in tumor-related tissues [44]. These genes are associated with glioma development, due to the activity of DNA-5-hydroxymethylcytosine (5hmC). Recent evidence has revealed that 5hmC is negatively correlated with the tumor level. Moreover, p16INK4a [45], p14ARF MLH1 [46], and NDRG2 [46] are other tumor-repressor genes that are correlated with glioma. The p16INK4a gene keeps the retinoblastoma tumor-suppressor protein (pRb) in the active form (dephosphorylated) in the normal cyclinD-Rb pathway. The active form of this protein controls the cell cycle’s progress [45]. GBM tissue shows a high incidence (greater than 50%) of deletion for the p16INK4a homozygous gene. Moreover, p16INK4a is changed in 80% of glioma cell lines. Thus, restoring the p16INK4a gene could decrease growth arrest and eliminate proliferation.

Steller et al. reported the hypermethylation of the MGMT promoter in approximately 40% of glioma tissues. The rate of methylation levels is dependent upon the zone in which the cells thereof have been cancerous [47]. Furthermore, the level of MGMT methylation is the critical index to determine TMZ susceptibility in glioma treatment. Low-adjusted MGMT can dramatically restore the in vitro and in vivo chemical sensitivity of TMZ [48]. A recurrent point mutation in isocitrate dehydrogenase 1 (IDH1) is frequently seen in adult diffuse gliomas. Mutant IDH1 gliomas are classified as mutant IDH1-1p/19q-codel or mutant IDH1-noncodel, based on the deletion of 1p/19q chromosomal regions [49,50,51]. IDH1 is the main source of NADPH in the human brain. It also can be found in other tissues of the body [52]. Most low-grade scattered astrocytomas (with a 75 percent mutation rate), anaplastic astrocytomas (with a 66 percent mutation rate), oligodendroglioma, promyelocyte, and secondary sex polymorphism include mutations in their IDH1/2 methylation regulatory protein [53]. The platelet-derived growth factor receptor alpha (PDGFRA) is a protein-coding gene that can reduce the growth of IDH mutant astrocytoma cells via the employment of demethylation medicines. This leads to the restoration of the normal function of such proteins [54,55]. Moreover, these medicines have a noticeable linkage to changes in NF1 and PDGFRA\IDH1, thus providing a practical treatment method [56].

The MAPK pathway or its downstream effectors contribute to carcinogenesis and proliferation in many types of malignancies. They can be activated in juvenile gliomas as a result of NF1 and BRAF gene alterations [57]. In addition, BMP signaling is activated in HGG tumor cells in children [58]. Activin A receptor type I (ACVR1) encodes for the type I BMP receptor ALK2. Its *somatic mutations, which are found in about 25% of pediatric brainstem gliomas, trigger BMP pathway activation [59]. Changes in the signaling pathways caused by specific genetic abnormalities in gliomas are promising targets for developing novel targeted gene therapies [60].

### 2.2. miRNAs and Glioma

Some studies have recently revealed that miRNAs play a major role in the transcriptional control, growth, and proliferation of numerous tumor genes [61,62]. Therefore, miRNA-based personalized medicine and gene-editing techniques seem to be viable strategies in cancer therapy. Approximately half of the miRNA genes are thought to be found in glioma cancer genes or in their vulnerable locations. These miRNA genes can affect 3% of all glioma tumor genes and 30% of all coding genes [63]. A single miRNA can simultaneously alter 100 GBM-related mRNAs, and a single glioma mRNA can be controlled by one or more miRNAs [64].

**Figure 1:**
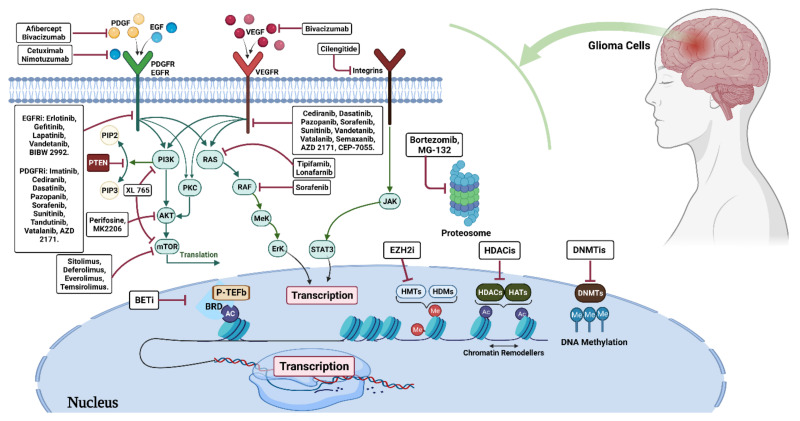
Schematic overview of molecules that have a vital role in GBM treatment. Growth factor receptors and intracellular signaling pathways can be activated in glioblastoma, thereby triggering gliomagenesis. Such pathways have the ability to induce tumor survival, invasiveness, proliferation, evading apoptosis, and avoiding immune surveillance [69]. EGF, VEGF, and PDGF, as well as the receptors thereof, can be blocked by small molecules and monoclonal antibodies [69]. Epigenetic process inhibitors, such as HDACis, DNMTis, BETis, and EZH2is, also have an important role to play in the treatment of patients suffering from GBM [69].

In the course of glioma disorders, miRNAs perform a number of important roles in carcinogenesis, the expression of cancer-related genes, glioma stem cell development, and regulatory pathways [65,66]. MiR-221/222 was discovered to be positively linked with the level of glioma cell invasion and infiltration. Zhang C. et al. have shown that the knockdown of MiR-221/222 could reduce cell invasion by manipulating the levels of the TIMP3 target. In a xenograft model, the knockdown of MiR-221/222 has enhanced TIMP3 expression and significantly decreased tumor growth [67]. Moreover, the overexpression of miR221/222 lowers the p27kipl levels and vice versa, which inhibits tumor proliferation (Figure 1) [68].

### 2.3. Chromatin Remodeling and Glioma

Mutations in the critical proteins of the regeneration complex are frequently found in human diseases [70]. These mutations are caused by aberrant chromatin remodeling [71,72,73]. Deficient chromatin remodeling can result in failed chromatin regeneration, in which the nucleosomes are unable to orient themselves properly, and the primary transcription machinery is prevented from acting [74,75,76]. These changes lead to aberrant gene expression [76]. Cancer develops when these mutations cause anomalies in tumor suppressor genes or cell-cycle regulatory proteins [77,78]. Drug-resistant tumor cells can be eliminated by targeting epigenetic and evolutionary processes [79,80]. This elimination could lead to avoiding disease recurrence [81,82]. Liu et al. have reported that targeting the GBM drug resistance stem cells (GSC) by kinase inhibitors can revert them to a slow-cycling, long-lasting state [81,82]. The notch signaling pathway is active in this state, and the histone demethylase KDM6A/B is considerably up-regulated [68,83]. This condition results in the removal of H3K27 trimethylation in the cis-regulatory region of the genome and higher levels of H3K27Ac [68,80]. This cellular transition was reported to be aided by chromatin remodeling [68]. The results of this study have identified a new target to develop effective anti-GBM treatments [84]. Moreover, according to a recent study [85], the expression of the controlled transcription factors E2F1 and GSK3 in astrocytomas and GBM was linked to glioma progression and LSH expression [83,85].

Glioma tissue also expresses the lipoprotein receptor protein (LRP6) 6, which is an upstream regulator of the GSK3 signaling cascade. LRP6 depletion decreases LSH expression by lowering the E2F1 engagement in the LSH promoter. These changes result in the suppression of cell growth. In light of these observations, the existing mechanical relationship between the activation of the LPR6/GSK3/E2F1 axis and LSH expression in glioblastoma could be interpreted as a key role in GBM. Understanding the role of LSH in the formation of gliomas adds to our understanding of the disease and makes LSH a possible therapeutic candidate to treat these lethal brain malignancies [86,87,88].

### 2.4. Histone Modification and Gliomas

Glioma formation and progression can be accelerated by improper histone modifications [89]. These aberrant modifications could lead to transcriptional irregularities and changes in the expression of enzymes, including histone methyltransferases, histone deacetylases (HDACs), and acetyltransferases (HATs) [90,91]. Histone methyltransferases are the histone-modifying proteins that have garnered the most attention [92]. Histone methylation at the lysine and arginine residues of the N-terminal region occurs on the H3 and H4 histones and is regulated by histone methyltransferases [93,94,95]. The main role of histone methylation is that of transcriptional regulation [96,97]. It could either repress the transcription through H3K9, H3K27, and H4K20 histone methylation or activate it via H3K4 histone methylation [98]. KMTs can be categorized into two protein families, based on their catalytic domain [99,100]. The two classes of KMTs comprise the SET domain-containing family (SUV39, SET1, SET2, SMYD, SUV4-20, SET7/9, and SET8) and the DOT1 family [99,101,102].

HATs participate in various biological processes, including cell-cycle progression, DNA damage repair, cellular senescence, and hormone signaling. HATs are among the bi-substrate enzymes that use the Ac-CoA cofactor and the histone lysine residue as substrates.

HDAC, HDAC1, HDAC2, HDAC3, HDAC5, and HDAC9 are among the HDACs that showed substantial alterations in glioma cells [103]. The histone acetylation of lysine residues in H2A, H2B, H3, and H4 typically drives gene transcription, while histone methylation can either activate (H3K4, H3K36, and H3K79) or repress (H3K9, H3K27, and H4K20) gene transcription [104]. HDAC5 and HDAC9 expression rise in high-grade medulloblastoma, compared to low-grade medulloblastoma and abnormal tissues [105].

## 3. Glioma Treatment Strategies

### 3.1. Glioma Epigenetic Therapy

#### 3.1.1. DNA Methylation Inhibitors (DNMTi)

The compressor tumor genes become silent by DNMT-mediated DNA methylation. Therefore, DNMT inhibition can restore the transcription of these important genes [106]. Thus, finding DNMT inhibitors could be deemed as a novel approach in glioma therapy. Aza-20-deoxycytidine 5 phosphate is the most common DNMT inhibitor, which is now in clinical trials. In tumor cells, aza-20 deoxycytidine-5 phosphate interacts with DNA and suppresses DNMT activity, which results in a favorable methylation state for anticancer activities [107].

#### 3.1.2. Histone Deacetylase Inhibitors (HDACi)

HDACis have been shown to suppress transcription and end cell division in G1 and G2 phases, promote cell differentiation and apoptosis, reverse the heat shock protein-substrate protein interaction, enhance oncoprotein degradation, and limit tumor growth and angiogenesis [108]. Individually or in combination, DNMT inhibitors and HDACis can be used to treat various tumors [109]. HDACi, as a novel therapeutic agent for glioblastoma, opens up new avenues for glioma treatment. Many HDACis have now entered stage I/II clinical studies to treat various kinds of glioma, including diffuse intrinsic pontine glioma (DIPG) and progressive or recurrent GB. HDACis can be used alone or in conjunction with other chemotherapeutic drugs, such as TMZ and radiation therapy [110]. 

Vorinostat and TMZ were tested in primary recurrent or refractory CNS malignancies, in a study conducted by the Department of Pediatric Oncology (COG) [111]. In children with recurrent CNS malignancies, five-day cycles of vorinostat in conjunction with TMZ were well tolerated. Vorinostat treatment resulted in the aggregation of acetylated H3 in peripheral blood mononuclear cells (PBMC). In a phase II study of verinavastat monotherapy, conducted by North Central Cancer Treatment, good tolerance in recurrent GBM patients was recorded. Moreover, an obvious increase in H2B and H4 acetylation levels was observed after treatment [112].

A phase II research study of Panobinostat, in conjunction with bevacizumab (BEV), in anaplastic glioma and recurrent GBM patients, was conducted [113]. Treatment was acceptable in both groups prior to closure. However, adding panobinostat to BEV did not increase the 6-month progression-free survival (PFS6) rate in either group when compared to BEV monotherapy controls. According to the additional preclinical studies, Panobinostat may serve as a sensitive agent. A phase-I study of stereotactic re-irradiation in combination with panobinostat has been reported in patients with recurrent HGG [114].

The COG has completed another phase of Valproic acid (VPA) research in children with refractory or CNS malignancies. At a steady state, half of the patients showed hyperacetylation of their histones. Krause et al. recently published a phase-II trial on simultaneous TMZ and VPA radiation therapy for GBM patients. Their findings revealed that the simultaneous administration of VPA and RT/TMZ is well tolerated in individuals with newly diagnosed GBM [115]. In general, HDACis appear to be promising therapies to improve the prognosis of malignant gliomas, when used as a monotherapy or combination therapy [116]. Improving or inventing novel epigenetic medicines or learning how to coordinate them with other treatments are viable options to reduce the damaging effect of epigenetic drug poisoning during treatment. HDAC, Vorinostat, and Valproic acid inhibitors have all had good clinical outcomes when paired with TMZ or RT in treating children with resistant or recurrent adult CNS or GBM cancers [115]. This could be a promising direction for future clinical investigations. Nonetheless, Vorinostat, in combination with erlotinib, or panobinostat in combination with BEV had no discernible effects [113]. Although these findings are not encouraging, they provide useful information for further studies.

Contemporary immunotherapy has become a popular strategy in cancer treatment. Even though few clinical trials on the combination of HDACis and the cytotoxic-mediated immunotherapy gene have been reported, most G-MCIs are recommended to be paired with TMZ. Most G-MCIs are recommended to be paired with TMZ. This strategy dramatically improved survival rates in individuals with minimal residual disease [117]. Tumor cells employ epigenetic processes to change autoimmune genesis and impair the process of tumor cell detection by the immune system. Tumor cells can kill themselves by lowering the expression levels of critical molecules in the tumor immune response process. They exploit DNA methylation or histone modification to exert these functions [118]. Immunosuppressive medications, such as cytokines and polypeptide vaccines, are currently the most common immunosuppressive drugs that can be applied in combination with epigenetic therapies [119,120]. This approach has become an increasingly popular strategy for the treatment of malignancies such as gliomas (Table 1).

## 4. Histone Methyltransferase

The enhancer of zeste homolog 2 (EZH2) is an H3K27 methyltransferase that is overexpressed or mutated in numerous human malignancies, such as gliomas [179]. Overexpression of EZH2 is linked to insignificant prognosis in GBM [180]. UNC1999 and GSK343 are two EZH2 inhibitors (EZH2i) that can inhibit the growth of GBM [179]. This capacity suggests that EZH2i could be used for GBM treatment [181,182]. 

Stazi et al. have developed and tested two novel EZH2i (MC4040 and MC4041) to evaluate their effects on primary GBM cell cultures [179]. MC4040 and MC4041 inhibited EZH2 at single-digit micromolar concentrations, with 10-fold lower effectiveness against EZH1 [183]. They have been shown to have no effects on other MTs. They have lowered the H3K27me3 levels in primary GBM cells and U-87 GBM cells [184]. These EZH2is have compromised GBM cell survival in a dose- and time-dependent manner without triggering apoptosis or interrupting the cell cycle in the G0/G1 phase, with elevated p21 and p27 levels [179]. MC4040 and MC4041 had higher impacts on cell survival when combined with TMZ [183]. Compared to MC4040 and MC4041, EZH2i tazemetostat (either alone or in combination with TMZ) is equally effective in the inhibition of GBM cell growth [179,183]. At the molecular level, MC4040 and MC4041 inhibited cell migration and invasion by reducing VEGFR1/VEGF expression, reversing the epithelial-mesenchymal transition (EMT), and reversing the malignant cancer phenotype. The pro-inflammatory phenotype of GBM cells was similarly affected by treatment with either MC4040 or MC4041. These treatments have significantly decreased the TGF-, TNF-, and IL-6 levels and increased the level of anti-inflammatory cytokine IL-10 [179,181,182,185].

### 4.1. Methyltransferase Inhibitors

#### 4.1.1. Bromodomain Inhibitors (BDI)

Bromodomains (BRD) and extraterminals (BET) are reader proteins that regulate gene transcription and chromatin remodeling by binding to the acetylated lysines of histones [186]. Considering the effect of various BET inhibitors, such as BRD2, BRD3, and BRD4 in multiple hematological malignancies, they could be suggested as novel therapeutic moieties in the epigenetic treatment of glioma [187,188]. Cell cycle arrest and apoptosis were triggered by JQ-1, a BRD4 inhibitor. Despite the ongoing clinical or preclinical investigations of various agents, the obtained results indicate that glioma cells are resistant to BDIs. For example, although MK-8628 (OTX015) reached a phase-II clinical trial in recurrent GBM, the trial was terminated due to an observed lack of clinical activity [189].

#### 4.1.2. Arginine Methyltransferase (PRMT5)

The expression of protein arginine methyltransferase 5 (PRMT5) is negatively correlated with the survival rate of glioma patients. Although the attained results suggest an association between glioma growth and PRMT5 activity, only one phase-Ι study of the PRMT5 inhibitor (PRT811) has been registered to date [190].

### 4.2. Glioma Immunotherapy

New immunotherapy strategies are being investigated in various preclinical and clinical investigations. During the clinical trials, unexpected responses have been observed and CAR-T cells have shown promising outcomes. Further studies are imminently required to reach a conclusion regarding the routine application of immunotherapy in glioblastoma treatment [160]. In the following sections, we will discuss the role of various immunotherapeutic approaches in glioma.

#### 4.2.1. Immune Checkpoint Inhibition

In glioma, the therapeutic suppression of PD-L1, IDO, or CTLA-4 lowers the amount of tumor-infiltrating Treg cells and improves long-term permanence [191]. The blockade of safety checkpoints seems to offer encouraging results as a method in glioma immunotherapy (Table 2).

##### PD-L1

The activation of the programmed cell death ligand 1 (PD-L1) as an immunosuppressive molecule lowers the T lymphocyte function and allows cancer cells to bypass the immune system [191]. PD-L1 expression is associated with the degree of glioma in human glioma tissues [125]. The use of PD-L1-based immunotherapy in the treatment of melanoma and small-cell lung cancer has shown considerable clinical benefits in recent years [213,214]. The average life duration of the NK cells under PD-1 residency was extended to 44 days in a study that examined the natural killer cells suppressed by PD-1 (NK), utilizing animal models of orthotopic glioma (GSC) stem cells [215]. The NK cell treatment group had a 35-day survival rate, while the control group had a 29-day survival rate. PD-1 inhibition was found to boost the cytotoxicity of NK cells against GSC in this investigation. Another appealing strategy is to combine the PD-1 inhibitors with other medicines. The combination of anti-PD-1 immunotherapy with stereotactic radiosurgery has increased median life by 52 days in a mouse model implanted with GL261 cells, compared to radiation for just 27 days with anti-PD-1 immunotherapy for 30 days [126].

Furthermore, compared to other therapies, a combination of anti-PD-1 immunotherapy, anti-TIM-1 antibody, and SRS dramatically improved the OS of GBM mice [191]. Moreover, the combination of PD-1 and VEGF inhibitors has been confirmed as acceptable and promising in different animal models and clinical trials [126]. In addition, the monoclonal antibodies of GVAX (anti-PD-1) in combination with anti-OX40 were found to be highly effective against mouse cerebral glioma [126].

In conjunction with Ipilimumab, Nivolumab is used as an FDA-approved IgG4 monoclonal antibody against PD-1 for the first-line treatment of metastatic melanoma. In a third-phase clinical trial, the safety and efficiency of Nivolumab and Ipilimumab were assessed in patients with recurrent glioblastoma (NCT02017717). The obtained results revealed that overall survival would not be improved by the combination of Nivolumab and ipilimumab, compared to taking nivolumab as a monotherapy, which had far better results (10.4 vs. 9.2 months) [126].

The efficacy of the Nivolumab neoadjuvant in patients with glioblastoma was investigated in a second-phase single-arm clinical trial. The Nivolumab neoadjuvant had a therapeutic effect on local immunoregulatory therapy [126]. Pembrolizumab is a PD-1-specific human monoclonal antibody showing satisfactory clinical activity and immunity [126]. It has been demonstrated that using Pembrolizumab as a neoadjuvant improves both the local and systemic immune responses to malignancies. Patients who received Pembrolizumab alone had a median OS of 228.5 days, compared to 417 days in the neoadjuvant group [126].

Anti-PD-1 immunotherapy has been shown to have anti-tumor effects in mice models and clinical trials. Moreover, its combination with other immunotherapies gives novel treatment options for glioblastoma. ClinicalTrials.gov has reported the numerous trials that have been reported so far.

The regulatory effect of ICs on immune responses is an essential issue that should be taken into account [216]. Accumulating evidence indicates that inhibitory/stimulatory ICs play a vital role in the modulation of immunological responses [217]. These molecules are imperative for maintaining homeostasis in the body; nevertheless, the expression of inhibitory ICs, such as PD-L1, can protect the tumor cells against anti-tumor immune responses. Thus, the blockade of inhibitory ICs has received a great deal of attention in terms of the treatment of various malignancies [218]. It should be noted that PD-1 has been linked to activated anti-tumor immune responses in patients with cancer [219]. VISTA is a new inhibitory IC that is also known as the PD-1 homolog (PD-1H), SISP1, differentiation of embryonic stem cells 1 (Dies1), B7-H5, and C10orf54 [220]. VISTA is mostly expressed in the parenchyma resident microglia and macrophages in the central nervous system [221]. Microglia have been linked to the development of glioblastoma, due to their ability to express inhibitory ICs. According to the studies conducted by Flies et al., VISTA can limit the growth of CD4+ T and antigen-presenting cells, which is in line with their role in the development of glioblastoma. Furthermore, they discovered that VISTA knockout mice are less prone to glioma, compared to wild-type mice [222].

##### CTLA-4

CTLA-4 is an immunological checkpoint protein that suppresses T-cell-mediated immune responses [223]. Patients with glioma have been found to express CTLA-4, and this expression has been implicated in glioma progression [223]. In mouse models, reduced CD4 + FoxP3 + Treg cells were observed following anti-CTLA-4 antibody treatment. This reduction has led to glioma eradication and increased long-term survival [224]. Another study has found that IL-12 and anti-CTLA-4 antibodies work together [130].

Furthermore, a combination of anti-CTLA-4 and anti-PD-1 antibodies have destroyed 75 percent of glioblastomas in mice, compared to 50 percent and 15 percent for anti-PD-1 and anti-CTLA-4 antibodies, as a monotherapy approach [225]. The combination of anti-4BB anti-agonist antibodies, anti-CTLA-4 antibodies, and targeted irradiation has led to a 50 percent increase in tumor-infiltrating lymphocytes and a 50 percent increase in tumor-free survival [226]. It is worth noting that all of the preceding experiments used GL261 cells to establish a glioma mouse model. However, a newer study has found that the SB28 mouse model was far more suited for the evaluation of glioblastoma immunotherapy. The SB28 mouse model has a decreased level of MHC-1 pressure and moderate CD8 T cell penetration [227]. Phase I and II clinical studies of Ipilimumab, in combination with other medications, such as Nivolumab, Temozolomide, or radiation, are currently being assessed, owing to the promising outcomes of the anti-CTLA-4 immunotherapy (NCT02794883).

##### Indolamine 2,3-dioxygenase (IDO)

Indolamine 2,3-dioxygenase (IDO) is an inhibitory and rate-limiting enzyme that is involved in tryptophan degradation. It regulates immunity by lowering T cell activity [228]. IDO activation may assist immune cells in evading immune surveillance during the development of malignancies. According to a recent, thorough investigation and meta-analysis, increased IDO1 expression was linked to poor prognosis in a variety of cancers, including glioma [229]. In addition, IDO inhibition, combined with chemotherapy, extends the lifespan of glioblastoma mice.

IDO inhibition could lead to higher C3 supplementation via chemotherapy at the tumor site and, ultimately, to immune-mediated tumor death [230]. The reduction of tumor cell proliferation by CD3+, CD4+, and CD8+ T3 cells could increase the survival rate and reduce tumor development in mice with glioblastoma [129]. This may be correlated with the propagation of CD3+, CD4+, and CD8+ T3 cells, which inhibit tumor cell growth [231]. In preclinical trials, IDO inhibitors appear to show tolerable efficacy. However, a phase-III clinical trial in melanoma patients has found that IDO inhibitors did not increase PFS or OS when compared to Pembrolizumab monotherapy [232,233].

#### 4.2.2. Bispecific Antibodies (BsAbs)

Bispecific antibodies (BsAbs) have recently demonstrated promising clinical effects in cancer immunotherapy. Hitherto, Removab [234], Blincyto, Hemlibra, and Rybrevant (EP2922872A1) have been the four marketed BsAbs, while several other BsAbs are in clinical trials [232,235]. T-cell-engaging bispecific antibodies (TCB), which direct and activate the circulating T cells against tumor locations, have sparked a great deal of interest in the scientific community [236]. Sun et al. have reported the desired results regarding GBM treatment using a BsAb, which was created using the “BAPTS” approach. LC/MS, SEC-HPLC, and SPR were used to characterize the BsAb. The other results showed that the EGFRvIII-BsAb has killed the EGFRvIII-positive GBM cells by attracting and activating the effector T cells that secrete toxic cytokines. The anticancer potential and long circulation time of EGFRvIII-BsAb have been demonstrated in NOD/SCID and BALB/c mice [237,238].

#### 4.2.3. CAR-T Treatment in Glioma

A domain that is often neglected in the assessment of chimeric antigen receptor (CAR) functionality is the extracellular spacer module. These molecules are endowed with the ability to detect special antigens and activate T cells [239]. The ectodomain detects a tumor-associated antigen expressed at the membrane of cancerous cells. The CAR endodomine comprises the stimulus molecules (CD28, OX40, CD137, and CD27) and CD3-derived intracellular T cell signaling domain [240]. Antigen-binding to the ectodomain triggers the transmission of an activation signal by the CD3 domain [241]. CAR-T cell therapy paved the way for a novel approach in cancer therapy; several CARs have been established against gliomas by targeting IL13Rα2, EGFRvIII, HER2, and CD70 [145]. It has previously been demonstrated that the intracranial delivery of first-generation CAR13 T-IL13Rα2 cells in patients with glioblastoma shows encouraging antitumor activity and is well tolerated [242]. This therapeutic strategy was examined in a phase-I clinical trial, based on a complete recovery report found in one patient [147]. The second generation of IL-1313 contains a CD137 stimulant and an imitative platform, which was included to enrich the central memory T cells. Compared to IL13Rα2, the effectiveness and durability of these CAR-T cells are enhanced [243].

In another study, the safety and cellular potential of HER2 CAR-T were examined. The phase I clinical trial results showed that HER2 CAR-T cells were well tolerated and presented clinical benefits in patients with glioblastoma [244]. Mostly, antigen escape during CAR-T cell therapy could cause tumor recurrence [148,245]. In addition, the intracerebral injection of EGFRvIII CAR-T cells can suppress tumor growth through IFN-γ secretion in the in vivo glioblastoma assays [246].

Targeting ICOS signaling as an anticancer strategy, in the case of EGFRvIII-expressing glioma, has been proven. CD70 was discovered to be a new immunosuppressive ligand in wild-type LGGs and glioblastoma [247]. Prior studies have also shown that CD70 expression was linked to a poor prognosis with glioblastoma. CD70 CAR-T cells could preferentially retract CD70 in xenograft mice, which confirms a new CAR target for glioma immunotherapy [248].

Numerous attempts have been made to find novel anti-glioma agents [69]. In mouse cancer models of breast, prostate, and lung melanomas, a CAR-T cell product targeting B7-H3 showed substantial anticancer efficacy. Furthermore, preclinical investigations have demonstrated that B7-H3 CAR-T cells have anticancer potential in GBM and pediatric malignancies [249]. These encouraging findings could pave the way for more clinical trials for GBM (NCT04077866) and juvenile glioma therapy (NCT04185038) [69].

In CAR-T cell-based treatments, Fc CR-T cells are also crucial. Fc CR-T cells, as with regular CAR-T cells, share a common intracellular tail as the stimulus domain. The Fc receptor (FcR), commonly known as CD16, replaces the extracellular CAR single-strand variable fragment. By targeting multiple tumor-associated antigens (TAAs), FcR can derive many monoclonal antibodies and drive NK cells to detect and kill tumor cells through antibody-dependent cytotoxicity (ADCC). As a result, Fc CR-T cells could be used as a novel cancer immunotherapy agent. However, the anticancer activity of Fc CR-T cells has not yet been confirmed in gliomas [250,251].

At present, there are some ongoing clinical trials that use CAR-T cells to treat glioblastoma. The majority of these CAR-T cells target EGFR, HER2, or IL13R2. However, some new targets have emerged, such as MUC1, GD2, CD147, and EphA2. The safety and anticancer efficacy of these CAR-T cells are being assessed in these studies. CAR-T cells can specifically target tumor cells, enhance the efficiency of treatment, and diminish the side effects. However, due to the production of diverse antigens and the restricted function of T cells at the tumor site, the therapeutic efficacy of CAR-T cells remains modest [69].

#### 4.2.4. Immunostimulatory Gene Therapy

The gene therapy approach could also be exploited in glioma treatment [252,253]. Whole genes and any regulatory elements may be delivered to the target cells of the patients by either mechanical methods or delivery vehicles [254,255]. Gene therapy vectors must be carefully selected to ensure the highest therapeutic efficacy [256]. To achieve the desired outcomes, therapeutic transgene expression levels, gene expression distribution within the TME, immunogenicity, and biosafety issues should be considered [256,257,258]. One beneficial feature of gene therapy is its ability to circumvent the BBB, which can be a problem for other systemic delivery techniques. Virotherapy is another promising treatment option for glioma. It involves using genetically modified viruses to kill tumor cells. The modified viruses of virotherapy are deprived of their ability to cause diseases and are able to replicate within tumor cells. The released oncolytic viral particles can infect and kill tumor cells in the surrounding area [252]. Different viral vectors, such as adenoviruses [250,252], retroviruses [132,259], and lentiviruses [133,260] have already been used in the gene therapy of GMB. In addition, non-viral vectors are gaining popularity for GBM gene therapy. Another major gene therapy approach is the use of different forms of RNAs, such as dsRNA, siRNA, or miR101 [252,261,262]. These RNAs, when used in combination with nanoparticulate systems, could increase GBM cell death [262,263]. The same nanoparticulate devices can also be used to restrict the growth and migration of GBM cells by targeting miR34 or proteins such as SOX9 and Ras [259,264,265].

##### Oncolytic Viruses in Gliomas

Oncolytic viruses (OVs) are a sort of immunotherapeutic agent that can infect and kill cancer cells. Newly proliferated viral particles are released once infected cancer cells die because of oncolysis. The released OVs would assist in the destruction of the remaining tumor cells [132]. Various oncolytic viruses have been studied in clinical and preclinical investigations for the treatment of GBM. OVs could also trigger immunogenic cell death (ICD), which results in anti-glioma immunity. 

ICD induces the production of tumor-associated antigens and immune-stimulatory molecules, which attracts the antigen-presenting cells (APCs) to the site of infection. Activated APCs ingest the tumor-associated antigens (TAAs) and detect the DAMPs via pattern recognition receptors (PRRs). Fully developed APCs travel to the lymph node to activate the anti-tumor cytotoxic CD8+ T lymphocytes (CTLs). Activated CTLs would lead to anti-glioma immunity. The recruitment of tumor-specific CTLs to the tumor site is derived from the viral-mediated production of type-I IFN and chemokine. Since the TAAs of glioma cells are displayed by their major histocompatibility complex (MHC) class I, CD8+ T lymphocytes can detect and kill them [252,260].

H101 is an oncolytic adenovirus that was approved in 2005 for treating head and neck cancer and metastatic melanoma [133]. Retroviruses, measles, and polioviruses are oncolytic viruses with promising anticancer activity [266]. Adenoviruses have been widely investigated due to their widespread use and ease of manipulation. They are non-enveloped, double-stranded DNA viruses with a number of advantages, including ease of genetic manipulation, high titers, low biosafety hazards, and a good safety profile following delivery into the brain. Deletion of the E1 and E3 regions of the first-generation AdV has rendered them non-replicative [267].

In mice with malignant gliomas, a combination of Ad5-24RGD and radiation therapy has shown great potential [268]. Immune responses that target tumor lysates are more likely to be involved in the obtained results. In addition, phase-I research (in an 8-year-old child with diffuse internal-portion glioma) has found no immunosuppression or harm from the virus [269]. However, follow-up studies are still needed (NCT03178032) to derive a conclusion. The combination of this oncolytic adenovirus with other therapeutic options, such as Pembrolizumab (NCT02798406), IFN-γ (NCT02197169), or TMZ (NCT01956734) is still under investigation. 

ONYX-015 is an oncolytic adenovirus with a weakened E1B protein, which is only capable of replication in p53-defective tumor cells. ONYX-015′s maximum tolerated dose is yet to be determined. However, increased immunity was linked to poor effectiveness in patients with malignant gliomas [270].

The use of HCV-derived oncolytic viruses is another promising strategy for glioblastoma treatment. R-115 is an oncolytic HSV that targets the human erbB-2 antigen. The combination of R-115 with IL-12 allows 30 percent of mice to clear tumors and substantially increased the middle OS [271]. Further clinical evaluations are required to understand the intricacies of this approach. G207 is another oncolytic HSV that is used in patients with malignant glioma and that is well tolerated in combination with radiation therapy. The intrauterine administration of G207 revealed an average survival rate of 7.5-months [272]. Two clinical trials are evaluating the safety and efficacy of G207 in children and adults with glioma (NCT00028158 and NCT03911388). M032 belongs to the second generation of oncolytic HSV. It can trigger tumor cells to produce IL-12 before their removal and can increase the efficacy of the treatment. M032 has been tested for its safety and tolerability in patients with GBM (NCT02062827). G47 belongs to the third generation of oncolytic HSV. The phase=II clinical trial of G47 (equipped with IL-12) was carried out in GBM patients receiving recurrent intratumoral stereotactic injections; the use of G47∆ may become a preferred treatment that potentially leads to the cure of malignant glioma in the near future [273]. 

The poliovirus receptor, CD155, is abundantly expressed in solid tumors. This receptor is targeted by the recombinant oncolytic poliovirus, PVSRIPO. Patients with GBM showed a 21 percent OS at 24 and 36 months after the intratumoral administration of PVSRIPO, while the historical controls showed a 14 percent OS at 24 months and a 4 percent OS at 36 months [274]. 

Lentiviruses (LVs) have also been widely examined for GBM therapy [275]. According to this approach, ablation of the TEA domain transcription factor 1 (TEAD1) could decrease human GBM cell movement and change the transcriptome signatures of migration and epithelial-mesenchymal transition (EMT) [276].

Vectors constructed on the Newcastle disease virus (NDV) have a natural affinity toward tumor cells and show oncolytic and immuno-stimulatory capabilities. Compared to the mono-therapy of TMZ, supplementary treatment with the naturally oncolytic NDV (the LaSota strain) could enhance apoptosis in glioma cells [277]. Besides, the combined therapy has dramatically increased the survival rate in a rat xenograft tumor model [278]. Finally, in vivo immunovirotherapy by measles virus (MV) strains, combined with anti-PD-L1 agents, improved the infiltration of activated CD8+ T cells and the survival of syngeneic GBM animals [279]. Oncolytic viruses have proven safe and effective when treating glioma in several investigations. Nonetheless, further research is required to see if oncolytic viruses can be used as a conventional glioma treatment.

##### Suicide Gene Therapy

The most studied gene therapy strategy for the treatment of glioma is suicide gene therapy. This strategy involves the expression of enzymes that transform a non-toxic prodrug into a cytotoxic drug. Furthermore, since this method is harmful to replicating cells, it preferentially targets the dividing tumor cells. These cells were produced due to the induction of pluripotent stem cells (hiPSCs). According to GBM xenograft models, the directed migration of these NS/PCs and the consequent tumor growth suppression could be detected [252,280].

##### Cytokine Therapy

The immune system produces cytokines as protective mediators to suppress immunological responses [137]. These effects, on the other hand, can be utilized to trigger immunological responses in tumor cells. Interleukins and interferons are the most commonly used cytokines in cancer treatment. IL-2 has been approved as an important growth factor for T cell activation in renal cell carcinoma and melanoma therapy [138]. The safety of IL-2 has been investigated in patients with glioma. However, the combination of systemic IL-2 and autologous tumor vaccination triggered significant therapy-related adverse effects [281]. As discussed before, combination therapy for the herpes simplex virus type 1 (HSV-TK) genes and the IL-2 coding genes was well tolerated in recurrent GBM patients with 50% tumor responses [282]. 

IL-4 is a glycoprotein that can assist T cells and cytotoxic T lymphocytes to proliferate and differentiate more quickly [282]. Adult HGG patients receiving autologous fibroblasts combined with HSV-TK genes and IL-4 coding genes have been shown to have a sufficient clinical response [139]. In a first-phase therapeutic trial, 31 patients with recurrent HGG were given a recombinant chimeric protein containing IL-4. There were few side effects, and no deaths were linked to the treatment [283]. In a phase-I study, the safety of a recombinant IL-13 protein and *Pseudomonas aeruginosa* exotoxin A, IL-13-PE38QQR, was validated in patients with recurrent HGG [284]. Furthermore, Phase-III research has found that patients’ PFS was extended following IL-13-PE38QQR therapy (17.7 vs. 11.4 weeks). However, there were no significant differences in the OS of the two groups [285]. IFN- has been licensed for the treatment of blood cancers, such as leukemia and lymphoma, and solid tumors, such as Kaposi’s melanoma and sarcoma [286]. IFN- has been demonstrated to have anticancer efficacy in preclinical trials. IFN- has also been shown to enhance the effect of TMZ in recurrent GBM patients in stage II experiments [287,288]. In preclinical investigations, IFN- has been reported to boost chemical sensitivity to TMZ via declining MGMT transcription. In addition, a first-phase clinical trial has found that adding IFN- to regular TMZ chemotherapy is well tolerated and may extend the lives of GBM patients [289]. 

The University of Nagoya conducted a multicenter clinical trial to treat patients with HGG using cationic liposomes and an IFN- expressing gene. The results showed that 40% of patients demonstrated a more than 50% decrease in tumor size. Moreover, the treated patients had a median survival rate that was higher than in the preceding control group [290]. In the case of GBM or HGG, the combination of IFN- and radiation therapy or chemoradiotherapy had no significant effect [291].

#### 4.2.5. Tumor-Associated Macrophage Treatment in Gliomas

TAMs (tumor-associated macrophages) are macrophages that reside in tiny habitats around tumors to speed up tumor progression [292,293]. Previous studies have revealed that non-suppressive M2 macrophages result in the establishment of an invasive milieu that aids glioma progression [294]. Microglia have a crucial role in the innate immunity of CNS-like macrophages [295]. Immunohistochemistry labeling has shown that tumor pathology, treatment response, and overall survival in glioma patients influence macrophage gene expression [296]. TGF-1 is secreted by activated microglia, which then invade the glioma. In addition, the co-culture of microglia and glioma cells has increased TGF-1 expression [297]. This finding was validated by another investigation, in which CD133+ glioma cells were shown to have higher TGF-1-induced invasive potential than CD133 cells [298]. 

Matrix metalloprotease 9 (MMP-9) has a significant role in glioma cell invasion. CCL2 expression is dramatically elevated in glioma U87 cells grown with microglia, which enhances glioma cell invasion. Anti-CCL-2 antibodies were reported to drastically reduce the infiltration of microglia in mice with glioma. Furthermore, a considerably extended OS was observed in mice with glioma, using a combination of anti-CCL2 antibody with temozolomide [1,299].

Since the differentiation and survival of TAMs are dependent on the colony-stimulating factor (CSF), a CSF-1 inhibitor (known as BLZ945) has been utilized to target TAMs in mouse glioma models. The attained results have revealed that the inhibition of CSF-1 can reduce the number of M2 macrophages in the TAM population. This reduced number of M2 macrophages could lead to improved tumor survival and regression [300]. In recurrent glioblastoma, however, similar therapeutic effects were not reported. In recurrent cancers, combining a PI3K or IGF-1 inhibitor with a CSF-1 inhibitor has dramatically extended the OS and has provided a novel approach for CSF-1 inhibitor resistance [301]. In the glioblastoma mouse model, PLX3397 can lower the amount of tumor-associated microglia and tumor invasion [302]. A second-phase clinical trial was carried out on 37 individuals with recurrent glioblastoma to assess the safety and efficacy of PLX3397 [303]. The obtained results have demonstrated that PLX3397 is well tolerated but has no therapeutic benefit, necessitating additional research to determine the efficacy of combination strategies [142]. Furthermore, cyclosporine treatment has been demonstrated to diminish angiogenesis by inhibiting microglia proliferation. Propentofylline, which is used to treat glioma, has shown similar results [304].

TAMs are implicated in developing and maintaining immune suppression, tumor cell migration, and increased angiogenesis in gliomas. Immunotherapy approaches targeting TAMs appear to hold great promise in the treatment of gliomas. The TAM blocking method has only been tested in rodent models and in small clinical studies. Moreover, FDA has not yet approved any TAM blocking agents. More studies are required to better understand the relationship between gliomas and TAMs (Table 1) [305].

### 4.3. Therapeutic Gene Vaccines for Gliomas

Anti-glioma vaccines have been developed in a number of studies for glioma treatment or the prevention of its recurrence after treatment [306,307]. Peptides, dendritic cells, DNA, RNA (particularly non-coding RNA), and viral vaccination vectors are among the anti-glioma vaccines being studied in phase I and II clinical trials [308,309,310,311,312,313]. Vaccination with protein moieties mainly boosts the humoral immune response, whereas fighting against cancer demands both cellular and humoral responses. Due to their capacity to induce the CD8 T-cells, DNA vaccines are being evaluated in tumor immunotherapy for certain conditions [314]. A crucial step in vaccine development is the selection of an appropriate tumor antigen [315,316,317]. In clinical trials, various tumor antigens have been studied for glioma patients.

#### 4.3.1. Peptides-Based Vaccine

Peptide-based vaccinations are less prone to have adverse consequences, such as the generation of tolerance. This is because of the fact that peptide-based vaccines are capable of predicting antigens [318]. Peptides, such as EGFRVIII or IDHR132H, are derived from mutated proteins that are solely expressed in tumors. These peptides have the potential benefit of tumor selectivity and a lower risk of triggering autoimmunity when compared to peptides taken from routinely overexpressed proteins in gliomas. In glioblastoma, only a few tumor antigens have been identified. Thus, vaccination trials in glioma patients, and prospective integration of vaccinations into the standard of care in this environment, may face challenges not seen before in the clinical management of brain tumor patients [319].

In recent years, peptide vaccines have garnered much attention. Tumor-associated peptides produced from non-mutated proteins are highly common among glioblastomas, and their appearance is assumed to be mostly due to unregulated signaling pathways [320]. Personalized cancer vaccines can be considered the patient-specific selection of tumor-associated peptides that do not exist abundantly in most glioblastomas. These peptides have a wide pattern of expression, potential immunogenicity, and the capacity to increase the likelihood of the prosperous induction of relevant immune responses. IMA950 is a peptide designed for the therapeutic vaccination of glioblastoma patients. In addition to the synthetic peptide of hepatitis B virus (IMA-HBV-001), this vaccine contains 11 tumor-associated peptides. The first step in the clinical studies of IMA950 was completed in 45 individuals with recently diagnosed glioblastoma, who received maintenance temozolomide [172]. 

Immunocompetent mouse models have also been used to test peptide-based vaccinations, either alone or in combination with medications targeted to overcome glioma-associated immunosuppression. For instance, an anti-transforming growth factor (TGF) antibody improved the efficacy of vaccination, in combination with two peptides derived from putative glioma-associated antigens, GARC-177–85 and EphA2671–679 [321].

Vaccines may be more effective in conjunction with molecularly targeted medicines [322]. Similarly, co-treatment with anti-cytotoxic T cell antigen 4 (CTLA4) antibodies has increased the effectiveness of a vaccine made up of irradiated GL-261 cells expressing GM-CSF [127,299,323].

#### 4.3.2. Dendritic Cell Vaccines

Dendritic cell vaccines (DCVs) are antigen-presenting cell (APC)-based vaccines that can elicit immune responses [324,325]. Immature DCs can be differentiated from CD14+ monocytes [324]. These DCs could then be loaded with tumor antigens, which are then injected back into the patient [324,326,327]. In 2010, the FDA approved the sole DCV-based therapy for the treatment of metastatic prostate cancer, Sipuleucel-T (PROVENGE; Dendreon) [308,324]. DCV has been shown to be relatively safe and has shown promising efficacy for glioma treatment. There are currently two second-stage trials registered on ClinicalTrials.gov for DCV-based glioma therapy. The use of DCV with tumor samples, hematopoietic stem cells, allogeneic stem cells, and cytotoxic lymphocytes as a first-line treatment in patients with GBM is still being explored in one trial (NCT01759810). Another trial involving GBM patients is now examining mRNA-transmitted DCV and comparing this treatment with adjuvant TMZ (NCT03548571). The results of the third major clinical trial clearly showed that DCVs are a realistic and effective treatment for patients with HGG [1]. There are various antigens that are loaded on DCs, as discussed below.

##### Tumor Peptide Loaded DCs

**Figure 2:**
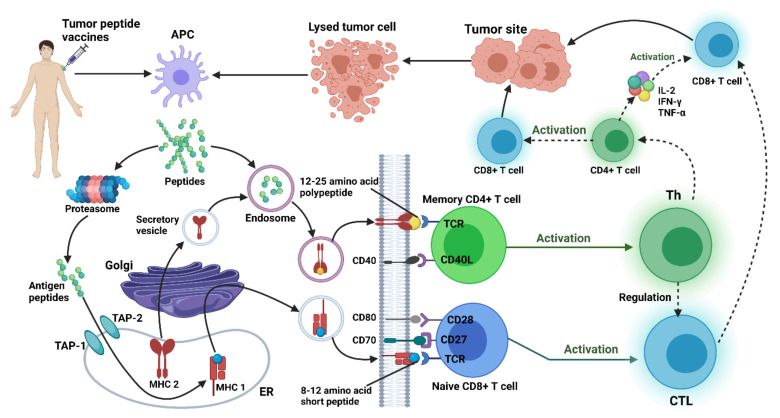
Tumor peptide vaccines have an immune system that activates T cells. Mature APCs may recognize and take up exogenous antigens when patients receive injections of the cancer peptide vaccines and the antigens enter the body. These are the main immunological reactions: (1) the proteasome converts proteins into small peptides that are sent to a transporter involved in the processing of antigens (TAP). The endoplasmic reticulum then binds 8–12 amino acid peptides to MHC–I (ER). (2) Following endocytosis, the antigenic polypeptides are broken down into endosomes. The resulting 12–25 amino acid peptides then interact with MHC–II, which was formed in the ER and discharged by the Golgi as vesicles. (3) The T cells become activated after recognizing and engaging the peptide-MHC complex via T cell receptors once the antigens are presented by APCs to lymphoid organs (TCRs). In the procedure, CD4+ T cells detect long polypeptides and the CD8+ T cells recognize short peptides. (4) Peptide-MHC complexes and costimulatory molecules operate as dual signals to activate immature CTLs from immature CD8+ T cells. To begin an efficient killing response, CTLs first travel to cancer locations. These cells produce a wide range of cytokines, including perforins and granzymes, as well as inflammatory mediators that dissolve target cells. (5) To maintain the expansion and proliferation of CD8+ T cells and support their antitumor effects, activated CD4+ T cells released cytokines such as IL-2, IFN-, and TNF. (6) Tumor antigens are further exposed by fragments released by tumor cell lysis, activating APCs and fostering antitumor immune responses.

DCVs with tumor-associated antigens have been extensively studied for their safety and efficacy as target peptides. EGFRvIII, as one of the earliest peptides loaded into DCs, has received the greatest clinical attention among the peptide vaccines. This peptide is either activated by the epidermal growth factor (EGF) or by transforming growth factor-alpha (TGF-alpha) [328,329]. After vaccination, the average progression and overall survival time were 6.8 and 18.7 months, respectively. In a clinical trial in individuals with recurrent HGG, DCV containing the IL-13R2, EphA2, gp100, and YKL-40 antigens indicated a dismal prognosis in vaccine-treated patients [330]. In a second-phase randomized experiment conducted in late 2019, the same DCV, known as ICT-107, showed substantial anticancer efficacy. In comparison to treated patients, 81 patients who received ICT-107 significantly improved the mean PFS (11.2 months vs. 9 months, respectively). Patients with HLA-A2 had strong clinical responses and immunity [331]. Only those patients with HLA-A1 methylation exhibited OS advantages (Figure 2) [159].

##### Tumor Lysate-Loaded DCs

Tumor lysate is made from autologous tumor cells collected upon surgical excision [178]. The patient’s tumor cells are lysed and then pumped into DCs, leading to the generation of cellular fragments. When DCs are exposed to these tumor fragments, they can phagocytose them and will present peptide antigens on the MHC class I and II molecules [332]. The use of tumor lysates has the advantage of the unbiased activation of DCs against the full complement of the patient’s specific TAAs. This approach could also unravel previously unknown tumor antigens [333]. DC-VaxL comprises autologous DCs, pulsed with autologous whole-tumor lysate, and is currently in a phase-III clinical study. It is one of the more well-established vaccination methods in this class [334]. The primary results of the Phase 3 clinical study of an autologous dendritic cell vaccination in newly diagnosed glioblastoma patients have demonstrated that adding DCVax-L to standard therapy is feasible and safe and may extend the survival rate [335].

Two phase I clinical trials were conducted to assess the safety of DCVs in patients with HGG. According to the attained outcomes, DCV employing autologous glioma cells was harmlessness and practical as the antigen [155,156]. In a second phase, a clinical investigation of GBM patients, along with their immune and clinical responses to DCV, were observed, with 17 of 34 patients achieving significant improvements in median survival (642 vs. 430 days) [309]. In 45 children with various CNS tumors (including 32 HGG), DCV was administered using tumor lysates as antigens [336]. The average survival for HGG patients was 13.5 months, while four out of twenty-two GBM patients lived for more than 24 months, according to the findings. This DCV approach was also employed in patients who had recently been diagnosed with GBM [337]. However, another trial reported no effects that enrolled newly diagnosed GBM patients treated with DCV tumors.

A second-phase clinical trial using DCV tumor lysates in patients with recurrent GBM found no significant difference in survival rates [338]. A trial of 11 patients with HGG experienced a partial response, and one patient received a complete response. The antigen is transformed into keyhole hemocyanin, which was discovered in a preliminary clinical trial [339]. The mean operating time in adults with newly diagnosed GBM was 22.8 months. In the last years, two second-phase clinical trials using DCV have yielded significant results [319]. Chang et al. have performed one of these trials with 19 HGG irradiated patients using DCV activated by tumor cells [340].

##### mRNA-Loaded DCs

There have also been other investigations using transfected mRNA. Unlike the tumor protein, mRNA may be amplified in vitro, providing a practical advantage with this approach. Amplification allows for the generation of a multiple antigen-loaded DCs from a small cell source [341,342]. 

Seven patients with GBM received a DCV, which was primed against transfected tumor mRNA, that it had a median OS of 759 days, compared to 585 days [343]. A Combination of a pp65-transfected DCV with GM-CSF and dose-intensified TMZ was employed in another trial to treat patients with newly diagnosed GBM. The median OS time for eleven patients was 41.1 months, compared to 19.2 months [344]. Several clinical trials are currently implementing this technique (NCT02649582, NCT02709616).

##### Glioma Stem Cell-Loaded DCs

Glioma cancer stem cells (GSCs) are a cellular subtype that is distinct from the rest of the tumor cell mass and has the ability to self-renewal [345]. Chemoradiation-resistant GSCs are thought to be the cause of recurrence [35]. In the phase I/II trial, Vik-Mo et al. studied seven patients who received a DC vaccination that targets stem cells (NCT00846456) [343]. In this regard, GBM specimens were dissociated, and patient stem cells were extracted and grown in vitro. The mRNA from stem cells was then amplified and injected into DCs. There were no serious side effects in the patients who received the vaccine. Furthermore, the PFS interval for treated patients was 2.9 times longer than controls (694 vs. 236 days, respectively). Recently in recurrent GBM, a phase I trial is assessing the safety of ICT-121, a DC-based vaccination targeting CD133+ (a stem-cell marker) cells (NCT02049489) [329].

##### Viral Antigen-Loaded DCs

**Figure 3:**
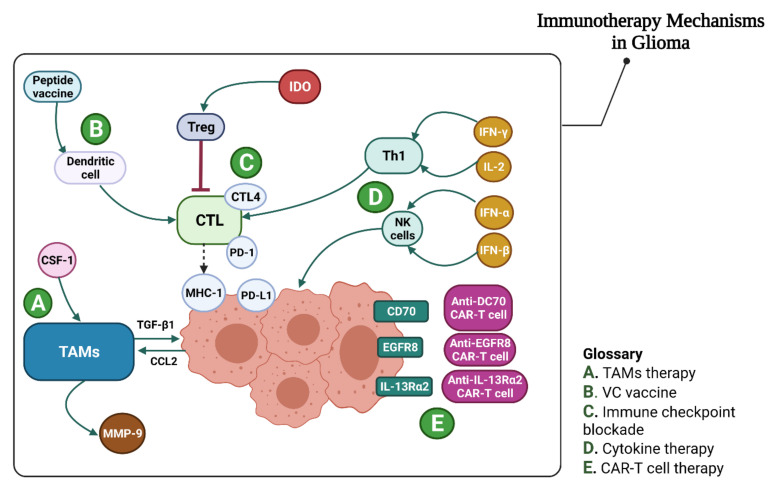
Current strategies for immunotherapy in glioma. DCs have a direct linkage to activating CTLs and to antigen presentation, thus killing glioma cells. PD-1, CTLA-4, and IDO as immune checkpoint ligands can be activated, which in turn lead to glioma cells escaping from immune surveillance.

In GBM, the presence of human cytomegalovirus (CMV) and its gene products are thought to play a role in the pro-oncogenic pathways [344]. CMV pp65 mRNA-loaded DCs in patients with glioblastoma (GBM) is being investigated (NCT00626483) [345]. A vaccine site with a recall antigen such as tetanus/diphtheria (Td) toxoid is capable of remarkably improving lymph node homing. In a blinded clinical trial, prior to immunization with DCs pulsed with pp65 RNA, 12 patients with GBM were randomized to undergo preconditioning with mature DCs or Td toxoid. The scientists found that patients who were pretreated with Td toxoid had improved DC migration and survival (Figure 3) [346].

### 4.4. Peptides-Based Treatment in Glioma

Peptides are short protein sequences that are formed from natural or synthetic sources and are typically less than 40 amino acids in length [347]. In addition to peptide vaccines, peptide-based treatments have wide applications in glioma therapy [348,349]. These treatments could be classified into three categories (Table 2) [350,351].

Tumor-targeting peptides are effective moieties to target tumor-specific receptors selectively [352,353]. Tumor-targeting peptides have higher tumor/tissue penetration than antibodies and are easier to produce and chemically modify to increase stability and pharmacokinetic characteristics [204]. These peptides have been used in imaging, cancer diagnostics, and targeted medication delivery [349]. Furthermore, certain peptides may be engineered to destroy cancer cells and stop tumors from progressing [352]. Tumor homing peptides can bind to overexpressed molecules of the cell surface in cancer cells [254,354]. Peptides that target abnormal cellular signaling pathways can reduce cancer cell activity, evasion from apoptosis, and proliferation [355]. Thus, more efficient peptides or their derivatives can be included in drug delivery systems for effective tumor targeting [356,357].

Cell-penetrating peptides (CPPs) can be produced from different origins that pass through the cell membrane to target the cancer cells specifically [358]. These short peptides can be covalently attached to a variety of drug carriers or medicines, such as oligonucleotides, imaging agents, various nanoparticles, etc. [359,360].

## 5. Future Directions

Notwithstanding incremental improvements in the treatment of glioblastoma, 5-year survival rates are still only at 10% [361]. There has been significant research exploring novel approaches in fields such as immunotherapy and precision oncology because there is an obvious need for better therapeutic strategies. This is motivated by a deeper comprehension of the molecular biology of glioblastoma and how it interacts with the immune system. Biological barriers such as the BBB and the distinct tumor and immunological milieu, in contrast to other solid tumors, provide substantial obstacles to the development of innovative treatments.

An emerging area of tumor therapy is immunotherapy. Different immune checkpoint inhibitors have shown promising results in a number of malignancies. In both pre-clinical and clinical trials, the traditional checkpoints PD-1/PD-L1, CTLA4, TIM3, and others have made impressive strides.

Combination therapy is also suggested due to the intricacy of the tumor microenvironment and the modulation of the immune response, particularly when anti-PD-1 and anti-CTLA4 therapy together show promise efficacy on rGBM.

Given the molecular heterogeneity and tumor evolution caused by single-target therapy, it is advisable to implement therapies targeting multiple antigens or with antagonistic immunosuppressive cytokines. This is because single-target therapy causes recurrence and, subsequently, resistance to the initial treatment.

Additionally, dendritic cell (DC)-based vaccines have been developed, such as DCVax-L (Northwest Biotherapeutics Inc., Marylandcity, USA), which uses autologous tumor tissue to produce tumor antigens. The results of an early phase-3 trial combining adjuvant temozolomide with DCVax-L are promising [361]. There are numerous other vaccinations in early-stage clinical trials, with targets such as IDH1 or multi-peptide vaccines [361]. Another rapidly growing field of research is chimeric antigen receptor (CAR) T-cell therapy, which uses genetically altered T cells and has produced spectacular results in a single case. Early trial results suggest that on-target action may be accompanied by enhanced T-cell infiltration [147,362]. However, more information is needed to comprehend its possible efficacy.

## 6. Conclusions

Glioma—a frequent and lethal tumor of the brain—can be divided into four grades. The first two grades are known to be low-grade, while the last two are deemed high-grade [361]. To treat patients afflicted with glioma, many challenges are seen in the face of dated strategies—radiotherapy and chemotherapy [361]. Adverse effects, poor prognosis, and glioma resistance against radiation and chemotherapy are the epitome of such challenges. In addition, genetic and epigenetic aberrations have been strongly linked with the development and molecular pathology of malignant glioma. Nonetheless, these challenges can be solved by novel therapeutic methods that are based on immunotherapy, vaccination, epigenetics, viral vehicles, and peptide moieties.

Genetic and epigenetic aberrations include abnormal methylation, microRNA changes, abnormal DNA, discoloration and structural changes, DNA methylation (particularly O6-methylguanine-DNA methyltransferase (MGMT) methylation), and histone modifications (methylation, acetylation, phosphorylation, and other modifications) that play a pivotal role in the progression of GBM. It was revealed that the compressor tumor genes become silent by DNMT-mediated DNA methylation. Therefore, DNMT inhibitors—the most common of which is Aza-20-deoxycytidine 5 phosphate—are able to restore the transcription of these important genes, which is considered a new approach in glioma therapy. Likewise, HDACis, whether used alone or in conjunction with other chemotherapeutic drugs, such as TMZ and radiation therapy, have been shown to suppress transcription, end cell division in G1 and G2 phases, promote cell differentiation and apoptosis, reverse the heat shock protein-substrate protein interaction, enhance oncoprotein degradation, and limit tumor growth and angiogenesis. Hence, they pave the way for glioma treatment. Of note, decreasing the level of the TGF-, TNF-, and IL-6 and increasing the level of anti-inflammatory cytokine IL-10 can be triggered by UNC1999 and GSK343, which are two EZH2 inhibitors (EZH2i), thereby inhibiting the growth of GBM.

Furthermore, new immunotherapy strategies are represented by other methods that can be used to treat patients suffering from GBM. The therapeutic suppression of PD-L1, IDO, or CTLA-4 lowers the number of tumor-infiltrating Treg cells and improves long-term permanence, whereby a blockade of safety checkpoints can be considered an encouraging approach in glioma immunotherapy. Incidentally, in vivo anticancer activity and the pharmacokinetic (PK) characteristics of the BsAb were determined using immunodeficient NOD/SCID and BALB/c mouse models, thus demonstrating the anticancer potential and long circulation time of EGFRvIII-BsAb. Chimeric antigen receptors (CARs) also paved the way for a novel approach to cancer therapy; several CARs have been established against gliomas by targeting IL13Rα2, EGFRvIII, HER2, and CD70. Oncolytic viruses (OVs), a sort of immunotherapy agent in which cancer cells are infected and killed by viruses, have been studied in clinical and preclinical investigations to treat GBM. OVs could also trigger immunogenic cell death (ICD), resulting in anti-glioma immunity.

Gene therapy—which involves the use of genetic elements to prevent and cure diseases—has drawn scholarly attention when used as a beneficial method to treat patients suffering from glioma. To arrive at the desirable outcomes of therapeutic transgene expression levels, gene expression distribution within the TME, immunogenicity, and biosafety issues should be considered. A beneficial feature of gene therapy is its ability to circumvent the BBB, which can be a problem for systemic delivery techniques.

Peptides, dendritic cells, DNA, RNA (particularly non-coding RNA), and viral vaccination vectors are among the anti-glioma vaccines being studied in phase-I and -II clinical trials. Vaccination with protein moieties mostly boosts the humoral immune response. Due to the anti-glioma vaccines’ capacity to induce CD8 T-cells, DNA vaccines are being evaluated in tumor immunotherapy for certain conditions. Unlike vaccines for infectious diseases, these vaccines are intended to boost the immune system, detect antigens, and more effectively combat cancer cells. Vaccines may be more effective in conjunction with molecularly targeted medicines, resulting in a realistic and effective treatment for patients with glioma.

One of the most challenging aspects of cancer treatment is the selective delivery of therapeutic chemical entities into cancer cells. Peptides targeting aberration cellular signaling pathways are capable of reducing cancer cell activity, the evasion of apoptosis, and the proliferation thereof. Thus, more efficient peptides or their derivatives can be included in drug delivery systems for effective tumor targeting. This selective delivery ensures therapeutic efficiency while minimizing the detrimental effects on healthy tissues.

## Figures and Tables

**Table 1:** Treatment strategies for GBM therapy.

Standard Management			
Category	Therapy	Mechanisms of Action	References
**Current treatments**	Surgical resection	Removing the possible amount of tumor in almost all types of gliomas.	[121]
	Radiation	Using high-energy beams after surgery, mainly in high-grade gliomas.	[122]
	Chemotherapy with Temozolomide (TMZ)	Binding to the genome, preventing the tumor cell growth and division.	[123]
	Tumor-treating fields (TTF) with bevacizumab	Selectively using an electromagnetic field with bevacizumab targeting vascular endothelial growth factor (VEGF).	[124]
**Future Directions**			
	**Therapy *(Study Numbers)***	**Mechanisms of Action**	**References**
**Checkpoint inhibitors**	**Immune checkpoint inhibitors** Programmed cell death protein 1 (PD-1/CD279) Nivolumab (*NCT02335918*) Bevacizumab (*NCT03743662*) Pembrolizumab (*NCT03899857*) Cytotoxic T-lymphocyte-associated protein 4 (CTLA-4/ CD152) Ipilimumab (*NCT02311920*) Durvalumab (*NCT02794883*) Tremelimumab Indoleamine 2,3-dioxygenase (IDO)	Decreasing the level, and cumulation of regulatory T (Treg) cells Increasing the survivorship Promoting the immune-mediated tumor destruction	[125,126,127,128,129,130,131]
**Immunostimulatory gene therapy**	**Oncolytic viral (OV) therapy** G207 (*NCT00028158 and NCT03911388*) M032 (*NCT02062827*) sh-SirT1 lentivirus (genetically engineered Lentivirus) miR-100 lentivirus (genetically engineered Lentivirus) DNX-2401 (modified adenovirus) (*NCT02798406, NCT02197169*) PVSRIPO ONYX-015 (Genetically modified adenoviral vector) H-1PV (wild-type parvovirus) Toca 511(a retroviral OV based on the murine leukemia virus) (*NCT02414165*)	Inducing the secretion of IL-12 by tumor cells, increasing the antitumor efficiency of the therapy Targeting highly expressed receptors in solid tumors (e.g., CD155 by PVSRIPO). Replication in p53-deficient tumor cells (e.g., ONYX-015) Improving radiotherapeutic sensitivity by silencing SirT1 (e.g., sh-SirT1 lentivirus) Increasing sensitivity to chemotherapy (e.g., miR-100 lentivirus) Converting the prodrug,5-fluorocytosine, into the antimetabolite by cytosine deaminase leads to tumor cell death 9, e.g., toca 511) Damaging genome and attesting the cell cycle (e.g., H-1PV) Reducing the number of tumor-associated macrophages Increasing the survivorship	[132,133,134]
	**Suicide gene therapy** Ad-TK (genetically engineered Adenoviral vectors encoding HSV thymidine kinase) Ad-Flt3L	Introducing thymidine kinase into the tumor Converting ganciclovir into an active form Inducing apoptosis in dividing tumor cells and releasing tumor antigens	[135,136]
	**Cytokine therapy** (HSVTK) genes and IL-2/ 4-encoding genes of IL-4/13 and Pseudomonas exotoxin (IL-4-PE) IFN-β with TMZ	Prolonging the survival of patients	[137,138,139]
**Adjuvant therapy**	**Tumor-associated macrophages (TAMs) therapy** Anti-CCL-2 antibody BLZ945 PLX3397(*NCT01790503*) Minocycline(*NCT02272270, NCT01580969*) Cyclosporine (*NCT00003625*)	Formation and retention of tumor cell migration Promoting angiogenesis Increasing the survivorship Inhibition of colony-stimulating factor Reducing the amount of the M2 macrophage Debilitating the expression of microglial secreting matrix metalloproteinases	[140,141,142,143,144]
**Passive immunotherapy**	**Chimeric antigen receptors (CARs) therapy** EGFRvIII(*NCT03283631, NCT03389230*) IL-13Rα2 (*NCT02208362*) HER2 (*NCT01109095*) CD70 Cd147 (*NCT04045847*) GD2 (*NCT03252171*) EphA2(*NCT02575261*) B7-H3(*NCT04077866*)	Inhibition of tumor growth via IFN-γ and IL-2 Secretion Receding CD70+ glioblastoma in xenograft models	[145,146,147,148,149,150]
	**Antibodies** Daclizumab (selective antibody for the high-affinity IL-2Rα)	Decreasing the amount of Tregs without affecting the T cells	[151]
**Active immunotherapy**	**EGFRvIII-mediated vaccine** Rindopepimut(CDX-110) (*NCT00458601*) MAb806 (ABT-806) (*NCT01472003*)	Targeting EGFRvIII tumor and increasing the immune response	[152]
	**Tumor cell vaccines** Temozolomide HSPPC-96 (*NCT01814813*)	Increasing efficacy using autologous tumor lysates Activating DCs (e.g., heat shock protein chaperon gp96)	[153,154]
	**Dendritic cell vaccines (DCVs)** ICT-107 DCVax-L Fusions of DC and glioma cells DCVax-L + GBM Pvax Pp65-DCs + GM-CSF1	Inducing strong anti-tumor immunity by activation of NKT, CD4, and CD8 cells	[155,156,157]
**Epigenome therapy**	**Inhibitors of mutant IDH (mtIDHi)** IDH305 (*NCT02381886*) AG-221 (*NCT02273739*) AG-120 (Ivosidenib) (*NCT02073994*) DS-1001b (*NCT03030066*) etc. **EZH2 inhibitors (EZH2i)** Tazemetostat (NCT03155620) **DNA methylation inhibitors (DNMTi)** 5-Azacytidine (Vidaza) (NCT02223052) 5-Azacytidine (Vidaza) (NCT03206021) **Histone deacetylase inhibitors (HDACi)** Valproic acid (*NCT03243461*) Vorinostat (SAHA) (*NCT01189266*) Belinostat (*NCT02137759*) Panobinostat(LBH589) (*NCT02717455*) Et	Normalizing the function of α-ketoglutarate-dependent enzymes Inhibiting polycomb repressor complex 2 (PRC2) Inducing innate immune response by reactivating retroviruses Modulating histone acetylation marks	[158,159]
**Combinational therapy**	**Combinations of multiple checkpoint inhibitors** Anti-CTLA4 (ipilimumab, tremelimumab) and anti-PDL1 (nivolumab, durvalumab, pembrolizumab) monoclonal antibodies Nivolumab in combination with the anti-LAG3	Increasing the survivorship	[160,161]
	**Checkpoint inhibitors combined with other immunotherapies** Vaccination with GM-CSF-expressing glioma cells and treatment with anti-CTLA4 antibodies Nivolumab with and without DC vaccine therapy PD-1 antibody blockade in combination with CD28-targeted CAR T cell Combination of a PD-1 inhibitor and VEGF inhibitor	Resulting in a higher antigen-specific immune response	[162,163,164,165]
	**Targeting immunosuppression in the tumor microenvironment** Combination therapy with the CD40 mAb and celecoxib Combination of a CD200R antagonist with tumor lysate vaccination	Activating myeloid cells toward a tumor-killing feature Inhibiting immunosuppressive functions	[166,167]
	**Combinations of multiple immunostimulatory gene therapies** Herpes simplex virus 1-thymidine kinase (HSV1-TK), and immune stimulation with Flt3L A cytokine that recruits DCs into the tumor microenvironment	Killing the remained proliferating tumor cells Releasing proinflammatory cytokines Stimulating DCs movement toward the tumor cells	[168]
	**Immunostimulatory gene therapy combined with other immunotherapies** Ad-TK/Flt3L in combination with subcutaneous vaccination with DCs, MDSC depletion with the anti-Gr-1 antibody following TK/Flt3L gene therapy PDL1 blockade combined with TK/Flt3L gene therapy CTLA-4 blockade combined with TK/Flt3L gene therapy	Increasing the survivorship	[169,170]
	**Vaccination combined with immune stimulatory adjuvants** Peptide vaccine containing 11 GBM-associated peptides, with GM-CSF Peptide vaccine containing 11 GBM-associated peptides, with poly-ICLC An agonist of TLR3 An IDH1 mutant peptide vaccine in combination with imiquimod (the TLR7 ligand) mTOR inhibition with rapamycin to enhance DC vaccine	Stimulating DCs maturation	[171,172]
	**Vaccination combined with other immunotherapies** Vaccination with CEApeptide pulsed DCs Anti-angiogenic therapy with immune-based therapies	Normalizing the tumor vasculature Increasing better infiltration of immune cells into the tumor cells	[173]
**Miscellaneous**	**Nanoparticle formulations** PLGA microparticles encapsulated with nicotinamide phosphoribosyltransferase inhibitor (GMX-1778) lipopolymeric NP NU-0129 (*NCT03020017*)	Selectively antagonizing NAD+ biosynthesis Targeting transcription factors, such as SOX2, OLIG2, SALL2, etc. Targeting Bcl-2-like protein by siRNA gold nanoparticles	[174,175]
	**BBB disruptive therapies** SonicCould (*NCT02253212*)	Using pulsed ultrasound to open the blood-brain barrier	[176]
	**Exosomes** miR-21sponge construct miR-199a miR-34a Long-noncoding RNA PTENP1 miR-146b	Inhibiting EGFR expression Compelling cell apoptosis Inhibiting cell proliferation and invasion	[177,178]

**Table 2:** Peptide-based treatment in glioma.

Peptide Name	Peptide Category	Sequence	Target	Carrier/Conjugate	Cargo/Drug	Ref
Angiopep-2 (ANG)	Tumor-homing peptides	TFFYGGSRGKRNNFKTEEY	Low density lipoprotein receptor	poly (ethylene glycol)-co-poly(ε-caprolactone) nanoparticles (PEG-PCL) copolymer nanoparticles (ANG-NP)	Paclitaxel (ANG1005)	[192,193]
Angiopep-2 (ANG)	Tumor-homing peptides	TFFYGGSRGKRNNFKTEEY	Low density lipoprotein receptor	Cell penetrating peptides: PepFect 14 (PF14) (PF14:TG1)	siRNA	[194]
Angiopep-2 (ANG)	Tumor-homing peptides	TFFYGGSRGKRNNFKTEEY	Low density lipoprotein receptor	Angiopep-conjugated liposome (ANG-CLP)	human tumor necrosis factor-related apoptosis-inducing ligand (pEGFP-hTRAIL) and paclitaxel (PTX)	[195,196]
Angiopep-2 (ANG)	Tumor-homing peptides	TFFYGGSRGKRNNFKTEEY	Low density lipoprotein receptor	PEGylated oxidized multi-walled carbon nanotubes (O-MWNTs)	doxorubicin	[197]
Angiopep-2 (ANG)	Tumor-homing peptides	TFFYGGSRGKRNNFKTEEY	Low density lipoprotein receptor	PAMAM dendrimers	Combination of Doxorubicin and borneol	[198]
Angiopep-2 (ANG)	Tumor-homing peptides	TFFYGGSRGKRNNFKTEEY	Low density lipoprotein receptor	Gold nanoparticles (PEG-DOX-AuNPs)	doxorubicin	[199]
Chlorotoxin	Tumor-homing peptides	MCMPCFTTDHQMARKCDDCCGGKGRGKCYGPQCLCR	Matrix metalloproteinase-2 (MMP-2)	nanovector	Green fluorescent protein (GFP) encoding DNA	[200]
Chlorotoxin	Tumor-homing peptides	MCMPCFTTDHQMARKCDDCCGGKGRGKCYGPQCLCR	Matrix metalloproteinase-2 (MMP-2)	Loaded liposomes	Doxorubicin	
Pep-1L	Tumor-homing peptides	ACGEMGWGWVRCGGSLCW	interleukin 13 receptor a2 (IL13Ra2)	Direct conjugation	Actinium-225, an alpha particle emitter	[201]
Pep-1L	Tumor-homing peptides	ACGEMGWGWVRCGGSLCW	interleukin 13 receptor a2 (IL13Ra2)	PEGylated nanoparticles	Paclitaxel	[202]
CooP	Tumor-homing peptides	CGLSGLGVA	FABP3/MDGI	PEG–PLA nanoparticles	paclitaxel	[203]
ACooPK	Tumor-homing peptides	ACGLSGLGVAK	FABP3/MDGI			[204]
T12	Tumor-homing peptides	THRPPMWSPVWP	Transferrin receptor (TRf)			
AP	Tumor-homing peptides	CRKRLDRNC	Interleukin-4 Receptor (IL-4R)	Gas (SF6)-filled microbubbles (MBs)	doxorubicin (Dox)-loaded nano-sized liposome	[205]
LP4	Peptides targeting aberrant cellular signaling pathways	SWTWEKKLETAVNLAW TAGNSNKWTWK	voltage-dependent anion channel 1 (VDAC1)	Cell-penetrating peptide Antp (Antp-LP4)	Direct inhibition	[206]
NBD	Peptides targeting aberrant cellular signaling pathways	TALDWSWLQTE	NEMO-binding domain (NBD)]	Cell-penetrating peptide Antp (Antp-LP4)	Direct inhibition of NF-kB	[207]
H1	Peptides targeting aberrant cellular signaling pathways	WPGSGNELKRAFAALRDQI	c-Myc	Elastin-like polypeptide (ELP) and penetrating peptide (Bac-ELP-H1)	Direct inhibition of c-Myc transcriptional activity	[208]
TAT peptide	Cell-penetrating peptides	GRKKRRQRRRPQ	Transactivator of transcription (TAT)	Polyamidoamine (PAMAM)	siRNA suppression of EGFR expression	[209]
TAT peptide	Cell-penetrating peptides	GRKKRRQRRRPQ	Transactivator of transcription (TAT) of human immunodeficiency virus	Liposomes	plasmid encoding green fluorescent protein (pEGFP-N1)	[210]
Syn B	Cell-penetrating peptides	RGGRLSYSRRRFSTSTGR	Antimicrobial peptide protegrin 1 (PG-1)	Direct conjugation	Doxorubicin Benzylpenicillin (B-Pc)	[211,212]
Penetratin peptide (Antp)	Cell-penetrating peptides	RQIKIWFQNRRMKWKK	Transcription factor antennapedia	Direct conjugation	Doxorubicin	[211]

## Data Availability

The datasets used and/or analyzed during the current study are available from the corresponding author, on reasonable request.

## Abbreviations

GBM	Glioblastoma
WHO	World Health Organization
OXPHOS	Oxidative phosphorylation
TMZ	Temozolomide
BBB	Blood-brain barrier
5hmC	5-hydroxymethylcytosine
IDH1	Isocitrate dehydrogenase 1
PDGFRA	Platelet-Derived Growth Factor Receptor Alpha
ACVR1	Activin A receptor type I
HDACs	Histone deacetylases
HATs	Acetyltransferases
DNMTi	DNA methylation inhibitors
PBMC	Peripheral blood mononuclear cells
PFS6	Progression-free survival
VPA	Valproic acid
EMT	Epithelial-mesenchymal transition
BRD	Bromodomain
IDO	Indolamine 2,3-dioxygenase
BsAbs	Bispecific antibodies
TAAs	Tumor-associated antigens
ADCC	Antibody-dependent cytotoxicity
PRRs	Pattern recognition receptors
HSV-TK	Herpes simplex virus type 1
DCVs	Dendritic cell vaccines
